# Craniofacial Pain Management in Severe COVID-19 Patients During the Pandemic Peak in Kosovo: A Comprehensive Approach

**DOI:** 10.7759/cureus.46111

**Published:** 2023-09-28

**Authors:** Valon Krasniqi, Visar Disha, Shaip Krasniqi, Merita Qorolli, Samire Beqaj

**Affiliations:** 1 Faculty of Medicine, Institute of Pharmacology With Toxicology and Clinical Pharmacology, University of Prishtina “Hasan Prishtina”, Prishtina, ALB; 2 Faculty of Medicine, Department of Prosthetic, University of Prishtina “Hasan Prishtina”, Prishtina, ALB; 3 Faculty of Medicine, Department of Physiotherapy, University of Prishtina “Hasan Prishtina”, Prishtina, ALB

**Keywords:** telemedicine, pain management, toothache, headache, covid-19

## Abstract

Background

This cross-sectional study aims to evaluate pain management's success in limiting admissions and assesses remote care's effectiveness for optimal pain relief. By utilizing data from severe COVID-19 inpatients in Prishtina, Kosovo, this study offers insights into the challenges posed by the pandemic and innovative care approaches aimed at improving patient well-being.

Methodology

This cross-sectional study includes 55 patients with severe COVID-19 after hospital discharge. All study participants completed the questionnaire in the presence of a clinical pharmacologist. The questionnaire of this study consisted of three parts: sociodemographic data (first part), the Intensity and Characteristics of Toothache (IaCofT) and headache (second part), and pharmacological treatment of headache and dental pain (third part). The questionnaire on IaCofT and headache was created with some modifications of the Modified Dental Pain Screening Questionnaire (M-DePaQ).

Descriptive statistics were conducted using Prism version 10.0.1 (Windows and Mac).

Results

According to the study data, 89.1% (*n* = 49) of the participants experienced pain during hospitalization with COVID-19, while 72.72% (*n *= 40) of them experienced pain after hospital discharge. Of the participants, 32.7% (*n* = 18) experienced dental pain, whereas 60% (n = 33) reported having headaches. Regarding the pain scale, more than two-thirds (*n *= 40, 72.72%) of the participants had moderate to moderately severe pain, and in 70.9% (*n *= 39) of the cases, the pain occurred episodically. The absolute majority (*n *= 53, 96.4%) of study participants reported the use of analgesics for pain management. Paracetamol (*n *= 46, 83.6%) and ibuprofen (*n *= 14, 25.5%) were the most commonly used analgesics for pain management.

Conclusions

This study highlighted the prevalence of headache and acute dental pain in these patients. The majority of the study participants were convinced by the healthcare system and were highly dependent on pharmacological treatment for headaches and acute toothache during the COVID-19 peak. The study results showed that the pain was proven to be successfully treated pharmacologically with analgesics such as paracetamol, ibuprofen, and diclofenac. Telemedicine is expected to become an important healthcare practice in the post-COVID-19 era. Therefore, the introduction of this service could be considered.

## Introduction

The 2019 outbreak of COVID-19, caused by SARS-CoV-2, has posed significant challenges to health systems worldwide. As the virus continued to spread rapidly, physicians, dental teams, and patients faced an increased risk of transmission due to close proximity during medical and dental procedures and the formation of aerosols [1]. The rapid spread of SARS-CoV-2 required strict safety measures to prevent further transmission while maintaining care for patients with chronic pain [2]. Restricting nonurgent medical services could be a good public health strategy to control outbreaks of healthcare-associated infections (HAIs), especially in situations of medical overcapacity [3]. In response to this global pandemic, medical and dental services have been adopted in many countries, including Kosovo, to mitigate the risk of transmission of COVID-19. The pandemic led to emergency measures being adopted by the Ministry of Health (MOH) in Prishtina, Kosovo, restricting general medicine and dental services to urgent cases [4].

Telemedicine gained prominence as attempts were made to overcome service limitations. The Prishtina MOH introduced a 24/7 hotline for general medicine and dental consultation, ensuring remote access to professional help in emergencies. These advances in telecommunications allow remote management of patients, minimizing the number of in-person visits while ensuring timely care [5]. The study by Peng et al. showed that telemedicine offers cost-effective benefits in the management of chronic pain in many countries, although some regions are doing their best to ensure the necessary resources [6].

Nevertheless, some reports question telemedicine. Some disadvantages of telemedicine have been mentioned by Balestra: limitations in performing complete physical examinations, technical barriers, potential legal problems, and various regulatory obstacles [7]. According to Hwei and Octavius, regardless of its advantages, the use and access to telemedicine are still associated with disadvantages that hinder its use in clinical practice [8].

All these disadvantages have become less important in the COVID-19 pandemic, as face-to-face contact increases the risk of disease transmission.

During August-September 2021 of the COVID-19 pandemic peak in Kosovo, 38.8 deaths were recorded per 100,000 inhabitants [9].

Considering the unique challenges posed by the COVID-19 pandemic, a triage-based management strategy has been adopted to effectively limit patient admission and prioritize urgent cases [10].

The treatment of craniofacial pain has taken on new dimensions with the advent of the COVID-19 pandemic [11]. In this context, recently, there has been a report by Rizk et al. hypothesizing that neuropathic pain manifests as toothache caused by COVID-19 infection without affecting the anatomical structures of the tooth and surrounding tissues [12].

Before the pandemic, craniofacial pain was commonly addressed through traditional methods, including pharmacological interventions, physical therapy, and lifestyle adjustments [13,14]. However, during the pandemic, the landscape shifted. Lockdowns, remote work, and increased stress led to a surge in reported craniofacial pain cases [15]. Lately, virtual consultations, telemedicine, and tailored home-based exercises have become essential tools for managing pain while minimizing in-person interactions [16].

Pre-pandemic craniofacial pain management primarily revolved around established treatments, such as analgesics, anti-inflammatory medications, and hands-on therapies. These interventions aimed to alleviate symptoms and improve patients' overall quality of life [17].

The COVID-19 period highlights the importance of adaptive craniofacial pain management. Conventional methods remain valuable, but according to the study by Provenzano and Narouze, the pandemic has illuminated the importance of other approaches such as telemedicine [18].

Therefore, we investigated whether telemedicine is an alternative for pain management in patients with limited access to healthcare facilities and the structure of analgesics used. This study aims to evaluate the success of pain management in reducing admissions and to assess the effectiveness of remote treatment for optimal relief. By analyzing data from severe COVID-19 patients from Prishtina, Kosovo, the study offers insights into the challenges of the pandemic and innovative care approaches that will provide tailored strategies for improved patient well-being.

## Materials and methods

This cross-sectional study was conducted at the University Clinical Center of Kosovo as part of a project with approval number 1233 (May 11, 2021) of the Ethical and Professional Committee of the University Clinical Center of Kosovo. Our research was of a qualitative type, which included severe COVID-19 patients who sought emergency consultation during the peak of COVID-19 infection in the August-September 2021 period.

Participants

A total of 55 participants who were initially hospitalized with a severe form of COVID-19 were included in the study following their hospital discharge.

Inclusion and exclusion criteria

The key inclusion criteria were as follows: a confirmed COVID-19 diagnosis using real-time PCR, the need for respiratory support for at least three consecutive days, and a minimum hospitalization period of seven days. Patients with pre-existing chronic pain conditions confirmed before hospital admission for COVID-19 were excluded.

We used in-person interviews to collect the data.

The study questionnaire consisted of three parts:

1) Sociodemographic data: The first part of the questionnaire collected information on the participants' age, gender, educational level, and place of residency during the COVID-19 pandemic peak.

2) The Intensity and Characteristics of Toothache (IaCofT) and headache questionnaire used in this study (second part) was derived through modifications to the Modified Dental Pain Screening Questionnaire (M-DePaQ) [19,20].

3) Headache and toothache pharmacological treatment: The third part of the questionnaire included questions on pharmacological usage for pain relief during the most recent headache and toothache episode. Additional questions developed by the authors were included to assess the utilization of telemedicine and remote medical and dental consultations by the study participants.

Data analysis

The collected data were entered into Prism version 10.0.1 (Windows and Mac) for analysis. Descriptive statistics were used to summarize patient administrative data, symptoms, pain intensity, medication use, telediagnosis, and pharmacologic advice.

Pain

Numeric Rating Scale-11 (NRS-11), a pain evaluation tool that is straightforward and convenient for scoring pain severity (PS), was used in this retrospective cross-sectional study [21]. It comprises 11 numerical values, spanning from 0 to 10, denoting absence of pain to utmost conceivable pain respectively [22]. NRS-11 holds dependable and authentic status as a self-report gauge for measuring pain intensity across diverse groups.

Patient Characteristics

Analyzed data included gender, age, marital status, parental status, education, and residential area during the COVID-19 outbreak. Additionally, we analyzed confidence in the health system to diagnose COVID-19, any long-standing diseases, and overall health.

BIAS Resolved

To mitigate potential biases, the patient selection process relied on the list endorsed by the Council of Infectious Diseases specialists at the Infectious Disease Clinic. Moreover, the study focused exclusively on patients hospitalized at the clinic between August and September 2021, aiming to minimize bias and ensure a comprehensive assessment of the given timeframe.

## Results

Demographics, education, and trust in institutions

A total of 55 patients who had previously been hospitalized with a severe form of COVID-19 responded to the prepared questionnaire. According to Table 1, the average age of the participants in this research was 53.71 years (standard deviation = 13.83), of which the majority belonged to the age group 41-50 years (*n *= 18, 32.72%), followed by the age group 61-70 years (*n *= 14, 25.45%) in second place and age group 51-60 years (*n *= 8, 14.54%) in the third. The age group least identified in this research was 20-30 years (*n *= 3, 5.45%). As regards gender distribution, 52.7% (*n *= 29) of the participants were male and 47.3% were female (*n *= 26). Of the participants, 92.72% (*n *= 51) were from the Prishtina region (Prishtina city and its surroundings) and most of them (*n *= 30, 54.5%) had completed high school. Of the participants, 89.1% (*n *= 49) were married and all of them had children. Less than half of the participants (*n *= 24, 43.63%) declared that in addition to COVID-19, they also had at least one chronic disease. In addition, 65.5% (*n *= 36) of the participants stated that they trusted health institutions a lot, while only 1.8% (*n *= 1) of them did not trust them at all.

**Table 1:** Demographic data of study participants. *n*, number of participants

Patient characteristics	Study modalities	n	Percentage (%)
Age (years)	20-30	3	5.45
	31-40	6	10.9
	41-50	18	32.72
	51-60	8	14.54
	61-70	14	25.45
	71-80	6	10.9
Gender	Male	29	52.7
	Female	26	47.3
Marital status	Married	49	89.1
	Not married	5	9.1
	Divorced	1	1.8
Parental status	With children	49	89.1
	Without children	6	10.9
Education	Master's degree	3	5.5
	Bachelor’s degree	8	14.5
	High school	30	54.5
	Elementary school	13	23.6
	No school	1	1.8
Residential area during the COVID-19 outbreak	Prishtina region	51	92.72
	Other regions of Kosovo	4	7.28
Trust in the health system to diagnose COVID-19	Very confident	36	65.5
	Somewhat confident	18	32.7
	Not at all confident	1	1.8
Long-standing disease	Yes	24	43.63
	No	31	56.4
Overall health	Excellent	9	16.4
	Good	42	76.4
	Bad	3	5.5
	Very bad	1	1.8

When we asked patients how they consider their health to be, 92.8% (*n *= 51) of the respondents stated that they are in good or excellent health and only 7.3% (*n *= 4) stated that they feel bad or very bad.

Intensity and characteristics of toothache and headache

Table 2 shows the intensity and characteristics of toothache and headache. The majority of respondents (*n *= 49, 89.1%) reported having pain during hospitalization with COVID-19, while 72.72% (*n* = 40) of them reported that they experienced pain again after being discharged from the hospital. About a third of the participants (*n *= 18, 32.7%) reported having a toothache. In addition, 60% (*n *= 33) of participants reported experiencing headache. Out of 55 participants, 11 (20%) reported both dental pain and headache. With regard to pain scale, 72.72% (*n *= 40) of the respondents reported that they suffered moderate to moderate-severe pain. In the majority of cases (*n *= 39, 70.9%), pain was episodic.

**Table 2:** The modified toothache and headache questionnaire. *n*, number of participants

Study questions	Answer modalities	n	Percentage (%)
Did you experience any kind of pain during the COVID-19 lockdown?	Yes	49	89.1
	No	6	10.9
Did you experience any kind of pain after discharge from the hospital?	Yes	40	72.7
	No	15	27.3
Did you experience toothache during the COVID-19 lockdown?	Yes	18	32.7
	No	37	67.3
Headache	Yes	33	60
	No	22	40
Toothache pain scale	Pain-free	2	3.63
	Mild	6	10.9
	Moderate	21	38.18
	Moderate to severe	19	34.54
	Extremely severe	7	12.72
Toothache and headache	Yes	11	20
	No	44	80
Pattern of pain	Episodic	39	70.9
	Continuous	16	29.1
Difficulty in sleeping	Yes	43	78.2
	No	12	21.8

An accompanying problem of the respondents was difficulty in sleeping. Of the participants, 78.2% (*n *= 43) reported facing such a problem.

Toothache and headache pharmacological treatment

We have presented the perception and patient's compliance regarding pharmacological treatment for toothache and headache in Table 3. The majority (*n *= 53, 96.4%) of the respondents who had experienced pain during COVID-19 reported the use of an analgesic for pain treatment. A large number of participants (*n *= 43, 78.2%) have bought the analgesic with a doctor's recommendation, while only 12.7% (*n *= 7) have bought the analgesic without the doctor’s prescription. The most used analgesic by the respondents was paracetamol (*n *= 46, 83.6%), followed by ibuprofen (*n *= 14, 25.5%) and diclofenac (*n *= 8, 14.5%). Most of the respondents (*n *= 39, 70.9%) stated that they used the pain medicine only when they experienced pain, while 18.2% (*n *= 10) of them used the analgesic every day. Concerning the degree of pain relief after drug administration, 41.8% (*n *= 23) of the respondents said that after taking the medicine, the pain was reduced by 26% to 50%; 38.2% (*n *= 21) reported that the pain was reduced by 51% to 75%, while only 5.5% (*n *= 3) of respondents said that the pain was reduced by 100%.

**Table 3:** Pharmacologic treatment of patients’ toothache and headache. *n*, number of participants; OTC, over the counter

Study questions	Answer modalities	n	Percentage (%)
Did you use analgesic for pain relief?	Yes	53	96.4
	No	2	3.6
Analgesic	a. Was recommended by a doctor/dentist	43	78.2
	b. Was recommended by a pharmacist at the pharmacy	5	9.1
	c. Was bought by the patient itself as an OTC drug	7	12.7
Analgesics used for treatment	a. Paracetamol (acetaminophen)	46	83.6
	b. Ibuprofen	14	25.5
	c. Diclofenac	8	14.5
	d. Naproxen	1	1.8
	e. Aspirin	10	18.2
	f. Nimesulide	1	1.8
	g. Ketoprofen	2	3.6
How often was the analgesic used?	Every day	10	18.2
	Several times a week	6	10.9
	Only when the pain was present	39	70.9
The percentage of pain reduction after analgesic use	a. <25%	8	14.5
	b. 26%-50%	23	41.8
	c. 51%-75%	21	38.2
	d. 76%-100%	3	5.5
Has your consultation with the doctor/dentist regarding the use of pain medication been interrupted?	Yes	29	52.7
	No	26	47.3

Moreover, according to statistical analysis, a significant difference was found between the age group 41-50 years and the age group 20-30 years (*P*-value = 0.027; odds ratio [OR] 11.25) as well as the age group 31-40 years (*P*-value = 0.044; OR 5.4) regarding research participants' use of analgesic for pain relief while they were sick with COVID-19 and after being released from the hospital.

In the other questions, there was no significant difference observed between different age groups or between genders (Table 4).

**Table 4:** Logistic regression analysis of the study results. ^*^P-value < 0.05. CI, confidence interval; OR, odds ratio

Survey question	Demographic data	Analyzed category	P-value	OR	95% CI
Did you use analgesics for pain relief?	Gender (Reference male)	Female	0.85	0.93	0.43-1.98
	Age (years) (Reference 41-50 years) Education (Reference high school)	20-30	0.027^*^	11.25	1.31-96.39
		31-40	0.044^*^	5.4	1.04-27.92
		51-60	0.18	2.47	0.65-9.30
		61-70	0.32	1.89	0.53-6.61
		71-80	0.32	1.89	0.53-6.61
Analgesic was recommended by a doctor/dentist	Gender (Reference male)	Female	0.136	2.78	0.72-10.65
	Age (years) (Reference 41-50 years) Education (Reference high school)	20-30	0.09	6.5	0.72-58.35
		31-40	0.09	6.5	0.72-58.35
		51-60	0.36	2.0	0.44-8.97
		61-70	0.89	1.1	0.29-4.12
		71-80	0.36	2.0	0.44-8.97
Analgesic used for pain treatment (paracetamol)	Gender (Reference male)	Female	0.11	0.26	0.04-1.39
	Age (years) (Reference 41-50 years) Education (Reference high school)	20-30	0.06	7.95	0.90-69.82
		31-40	0.11	3.81	0.71-20.37
		51-60	0.46	1.67	0.44-6.84
		61-70	0.66	1.33	0.36-4.87
		71-80	0.42	1.75	0.44-6.84
Analgesic used for pain treatment (Ibuprofen)	Gender (Reference male)	Female	0.31	1.89	0.53-6.61
	Age (years) (Reference 41-50 years) Education (Reference high school)	20-30	0.14	9.35	0.47-182.66
		31-40	0.14	9.35	0.47-182.66
		51-60	0.23	4.0	0.41-38.34
		61-70	0.23	4.0	0.41-38.34
		71-80	0.47	1.92	0.32-11.47
How often was the analgesic used? (Response: Only when pain was present)	Gender (Reference male)	Female	0.8	1.16	0.24-2.09
	Age (years) (Reference 41-50 years) Education (Reference high school)	20-30	0.09	6.5	0.01-46.88
		31-40	0.19	3.13	0.0076-3.62
		51-60	0.36	2.0	0.0076-3.62
		61-70	0.89	1.09	0.0112-7.38
		71-80	0.19	3.13	0.0076-3.62
The percentage of pain reduction after analgesic use (26%-50%)	Gender (Reference male)	Female	0.53	0.71	0.364-3.73
	Age (years) (Reference 41-50 years) Education (Reference high school)	20-30	0.95	0.9	0.72-58.35
		31-40	0.25	0.16	0.57-17.12
		51-60	0.25	0.16	0.44-8.97
		61-70	0.45	0.29	0.29-4.12
		71-80	0.25	0.16	0.57-17.12
The percentage of pain reduction after analgesic use (51%-75%)	Gender (Reference male)	Female	0.6	1.33	0.44-3.98
	Age (years) (Reference 41-50 years) Education (Reference high school)	20-30	0.31	4.81	0.22-105.14
		31-40	0.9	0.89	0.11-6.80
		51-60	0.9	0.89	0.11-6.80
		61-70	0.62	1.85	0.15-21.70
		71-80	0.9	0.89	0.11-6.8

These data show that differences among toothache and headache patients are more pronounced between age groups than between gender categories.

## Discussion

In response to the COVID-19 pandemic, numerous countries, including the Republic of Kosovo, implemented unprecedented measures by suspending non-emergency medical and dental treatment [23]. The stringent measures imposed by the Government to restrict people's movement in response to the peak of the pandemic and the health issues encountered by patients with severe COVID-19 upon discharge from the hospital resulted in a constrained participant pool for this study [4]. Therefore, the number of participants was limited to only 55.

This extraordinary shift in medical and dental healthcare policy was aimed at mitigating the spread of the virus and protecting both patients and healthcare personnel.

At the height of the pandemic, toothache and headache were the most commonly reported symptoms in severe COVID-19 patients.

Regarding the severity of pain, in our study, the prevalence of moderate-to-severe pain was 72.72% (*n *= 40), while 78.2% (*n *= 43) of the participants were affected by sleep disturbances. Most of our study participants (*n *= 40, 72.7%) belonged to the age group of 41-70 years, and all of them were affected by a severe form of COVID-19.

In contrast, the study conducted by Jarwan et al. [24] found that 47.9% of respondents had moderate-to-severe pain, while 45% were affected by sleep disturbances. Perhaps the age and severity of the disease of our study participants are the main reasons for the significant difference in pain perception. On the other hand, the significant difference in sleep problems among our patients may be attributed to the fact that they were individuals who had been treated severely for COVID-19 and subsequently discharged from the hospital.

Our study found that 96.4% (*n *= 53) of participants used analgesics for toothache/headache treatment during the COVID-19 pandemic, reflecting a significant reliance on pharmacological management. Paracetamol (83.6%), ibuprofen (25.5%), and diclofenac (14.5%) were the most used analgesics in our study. Similarly, a study by Patel et al. [25], conducted among adults in Texas, USA, during the pandemic also showed that 83% of dental emergencies were managed conservatively through analgesics for pain control. In their study, most used analgesics were ibuprofen, paracetamol, and naproxen. According to the results of Rinott et al. for pain relief, 32% of study patients used paracetamol, while 22% used ibuprofen [26]. The reason for this discrepancy in paracetamol may be that patients in our country had almost free access to the drugs because of pandemic circumstances and raised safety concerns for ibuprofen use during COVID-19.

The results of this study show that health systems' confidence in diagnosing COVID-19 varies widely across regions. In Kosovo, participants expressed relatively high confidence (*n *= 36, 65.5%) in their health system's ability to diagnose the virus. In contrast, results from participants in Makkah, Saudi Arabia (2023) [24] showed a significantly low utilization rate of 10% for the diagnostic capabilities of the health system. This discrepancy could be due to education, cultural beliefs, health insurance, and income.

Our study found that 78.2% (*n *= 43) of participants sought treatment on the recommendation of a physician, suggesting that there was substantial use of medical help for acute dental pain or headache during the COVID-19 pandemic. In our study, the team consisted of physicians and dentists, which was also observed in a study by Suh [27]. This collaborative approach proved effective in managing dental emergencies and allowed for optimal pain management and infection control strategies. Both studies highlight the importance of interdisciplinary collaboration in dental health care during times of crisis and offer valuable insights for future emergency preparedness and patient care strategies. In contrast, participants in a study by Nasir et al. [28] and Sujan et al. [29] had access to their physicians at nearly similar rates of 25% and 28.9%, respectively. This discrepancy could be because the participants in our study were hospitalized with a severe form of COVID-19 and pain management after discharge was based on the physician’s recommendation based on current guidelines. Another reason for this discrepancy could be the high educational level of the majority of our study participants (70% had completed high school and had a bachelor's degree). In addition, the 24/4 telecommunication service of the MOH in Kosovo actively supported these patients, which could have an impact on the high adherence to physician recommendations. Nonetheless, we suggest that further research be conducted on this topic, focusing primarily on patients' adherence to treatment after discharge from the hospital.

The novelty of COVID-19 and the lack of similar studies make direct comparisons difficult. Unlike previous studies that did not include patient outcome data [3], our study highlights the value of telemedicine beyond routine consultations [5]. The triage-based emergency management strategy we proposed effectively balanced limiting patient admission with pain control and symptom relief. The combination of remote information and systematic follow-up proved beneficial in confirming treatment efficacy and reassuring patients. This approach is consistent with a recent report from the World Health Organization and holds promise for addressing future requirements for regulating patient flow in emergencies [30].

Limitations

One limitation of this study was the difficulty in recruiting a larger number of participants. In addition, there was difficulty in motivating participants to complete an electronic survey and the failure to include other forms of pain in the group of study participants. Furthermore, some study participants failed to properly complete documentation, leading to their exclusion from the study.

## Conclusions

This study showed the prevalence of headache and acute dental pain in severe COVID-19 patients. The majority of participants in our study were confident in the healthcare system and relied heavily on medical interventions to treat dental pain and headaches during COVID-19. Pain was shown to be successfully treated with analgesics such as acetaminophen, ibuprofen, and diclofenac.

The results of this study may be a starting point for a more comprehensive review of the existing medical approach to patients with dental pain and headaches and the increased benefits of telemedicine services. The introduction of telemedicine services may be particularly useful in improving patient care and addressing accessibility issues to professional care.

Telemedicine could be a useful alternative to pain management for patients who do not have physical access to healthcare facilities. It is recommended that the cost-benefit of telemedicine services for patients with headaches and dental pain be investigated in another specific study. The most commonly used analgesics were acetaminophen, ibuprofen, and diclofenac. This pattern of analgesics fits with pain management practices in other surveys.
